# Spondylolysis: A Narrative Review of Etiology, Diagnosis, and Management

**DOI:** 10.3390/ijerph23020153

**Published:** 2026-01-26

**Authors:** Vanessa Madden, Adam Ayoub, Jonathan Thomas, Ian Thomas

**Affiliations:** 1 Thomas Sports and Regenerative Orthopedics (TSARO), Battle Creek, MI 49014, USA; 2 Homer Stryker Medical School, Western Michigan University, Kalamazoo, MI 49008, USA; adam.ayoub@wmed.edu

**Keywords:** spondylolysis, spondylolisthesis, pars interarticularis, athletic injuries, adolescent, pediatrics

## Abstract

**Highlights:**

**Public health relevance—How does this work relate to a public health issue?**

- Low back pain is a prevalent condition in pediatric and adolescent populations, and spondylolysis represents a common structural etiology in youth engaged in organized sports.
- Patterns of delayed diagnosis and variability in evaluation across care settings reflect broader public health challenges related to the early identification and management of musculoskeletal conditions in young athletes.

**Public health significance—Why is this work of significance to public health?**

- This review consolidates current evidence on the epidemiology, risk factors, and diagnostic approaches to spondylolysis, contributing to a clearer understanding of how sport-related exposures influence disease occurrence.
- By summarizing both traditional and emerging diagnostic modalities, the work informs discussions on resource utilization, radiation exposure, and equity in access to appropriate musculoskeletal care.

**Public health implications—What are the key implications or messages for practitioners, policy makers, and/or researchers in public health?**

- Improved recognition of spondylolysis as a contributor to adolescent low back pain may support earlier evaluation and more consistent management strategies across healthcare settings.
- Further population-based and longitudinal research is needed to define sport-specific prevalence, evaluate prevention strategies, and inform evidence-based guidelines relevant to youth sports participation.

**Abstract:**

**Background:** Spondylolysis is a stress fracture of the pars interarticularis, most common in adolescents and athletes involved in sports requiring repetitive spinal loading, extension, and rotation. The condition is often underdiagnosed due to delays in presentation and diagnosis, particularly among non-orthopedic providers. **Aims:** This review aims to summarize the current understanding of spondylolysis, focusing on its etiology, diagnosis, management strategies, and identify gaps in research for future exploration. **Methods:** A structured literature search was conducted in PubMed to identify studies relevant to pediatric and adolescent spondylolysis, spondylosis, and spondylolisthesis, particularly in the context of athletic injuries. The initial search yielded 143 citations. Applying filters for English language publications within the past five years reduced this to 125 citations. Limiting to populations that were aged 18 years and under returned 50 studies. After screening the titles and abstracts, 12 non-specific or irrelevant articles (including letters to the editor) were excluded, leaving a final dataset of 38 articles for detailed review. In addition, foundational and landmark studies outside this window were included to provide historical and conceptual context, bringing the total evidence base to 50 papers. **Findings:** Spondylolysis most commonly affects the L5 vertebra, with a higher incidence in male athletes. Conservative treatments like physical therapy and bracing are effective, especially when initiated early. However, the efficacy of bracing remains debated, with limited evidence on long-term clinical benefits. Surgical intervention is considered for severe or non-responsive cases. Diagnostic methods, including CT and MRI, are preferred, with emerging techniques like ultrasound showing potential for non-ionizing, cost-effective, early detection. **Implications:** Early diagnosis and treatment are crucial for preventing progression to spondylolisthesis. While conservative treatments often yield favorable outcomes, more research is needed to compare the effectiveness of bracing and pharmacological interventions. Future studies should focus on long-term outcomes, cost-effective, non-ionizing diagnostic methods, and the role of emerging therapies like regenerative medicine. A multi-disciplinary approach is vital for optimal patient care, particularly in young athletes.

## 1. Introduction

Spondylolysis is a repetitive stress injury to the pars interarticularis typically due to a combination of repetitive loading, extension, and rotation forces [1]. The injury is more common in children and adolescents where the pars interarticularis represent the most vulnerable portion of the spine that connects the facet joints to the spine. The incidence of spondylolysis in the general population ranges from 6 to 8%, with a male predominance [2,3,4].

Spondylolysis is more common in certain sports such as gymnastics, dance, football (particularly lineman), soccer, and basketball, with a prevalence ranging from 52 to 60% in relation to a pars interarticularis defect of any grade [5]. Despite having such a high prevalence in young athletes, one single-center retrospective study found that the average time from symptom onset to initial presentation was 24 weeks and from initial presentation to diagnosis was 15 weeks [6]. The study also found the delay in diagnosis took significantly longer for non-orthopedic providers (25 weeks vs. 1 week for orthopedic providers) and education on the diagnosis for primary care providers is warranted [6].

The purpose of the review is to review and summarize existing research on spondylolysis to examine the etiology, diagnosis, and management of the disease and highlight gaps in the existing data and potential areas for future research.

## 2. Methods

A structured literature search was conducted in PubMed to identify studies relevant to pediatric and adolescent spondylolysis, spondylosis, and spondylolisthesis, particularly in the context of athletic injuries. The following search terms were used:

(“Spondylolysis”[Mesh] OR spondylolysis OR “Spondylosis”[Mesh] OR spondylosis OR “Spondylolisthesis”[Mesh] OR spondylolisthesis OR olisthesis OR “Pars Interarticularis”) AND (“Athletic Injuries”[Mesh] OR athlet* OR sport*) AND (“Child”[Mesh] OR child* OR “Adolescent”[Mesh] OR adolescen* OR youth* OR teen* OR “Pediatrics”[Mesh] OR pediatric* OR pediatric* OR school* OR “young person” OR “young people”).

The initial search yielded 143 citations. Applying filters for English language publications within the past five years reduced this to 125 citations. Limiting to populations 18 years and under returned 50 studies. After screening titles and abstracts, 12 non-specific or irrelevant articles (including letters to the editor) were excluded, leaving a final dataset of 38 articles for detailed review. In addition, foundational and landmark studies outside this window were included to provide historical and conceptual context, bringing the total evidence base to 50 papers.

Studies were included if they involved the following:

1. They examined **spondylolysis**, **spondylosis**, **or spondylolisthesis** in pediatric or adolescent populations (≤18 years).
2. They reported on **sports-related prevalence**, **diagnostic strategies**, **management**, **or outcomes**.
3. They were original research studies, systematic reviews, or meta-analyses.

The exclusion criteria were as follows:

- Case reports or case series with <5 patients.
- Editorials, letters, or expert opinions without original data.
- Studies limited to adult populations without adolescent subgroup analysis.
- Non-English language publications.

**Figure 1:**
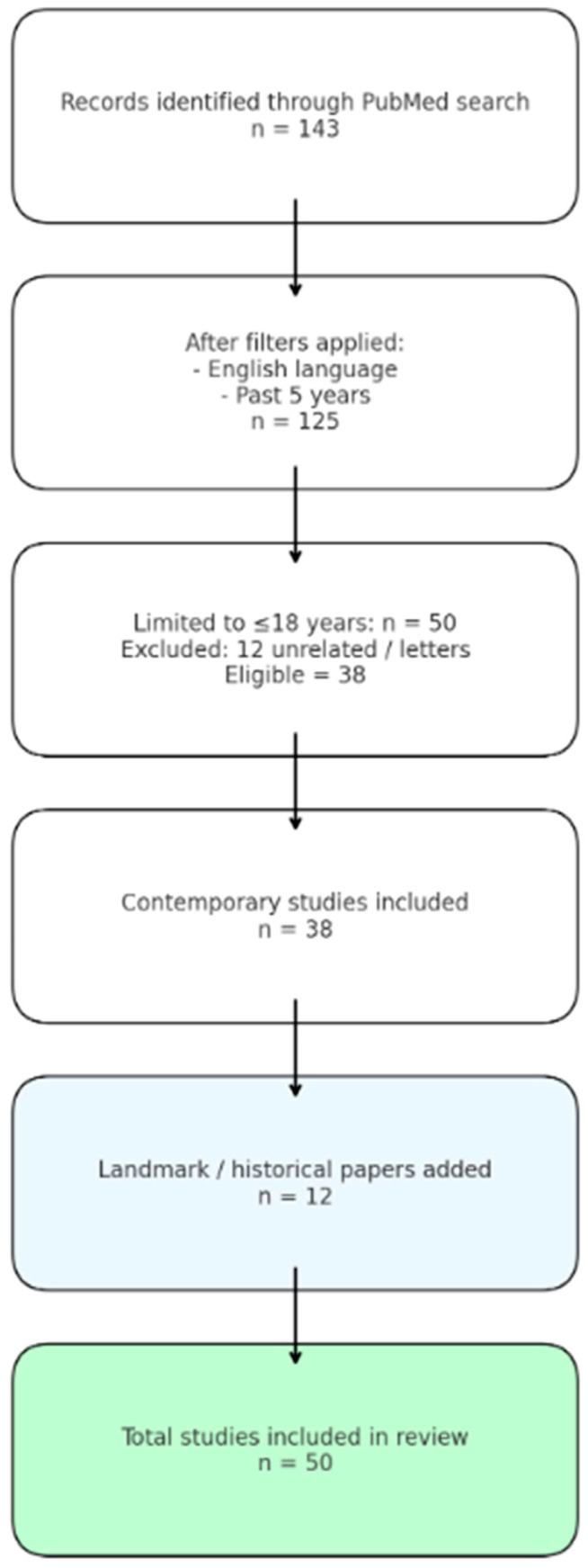
PRISMA flow diagram summarizing the literature search and study selection for pediatric and adolescent spondylolysis.

Screening was performed in two stages. Titles and abstracts were independently reviewed for eligibility, followed by full-text assessment of potentially relevant studies. Data extracted included study design, sample size, age and sex distribution, sport participation, diagnostic modality, management approach (conservative or surgical), and outcomes. To ensure comprehensiveness, both recent evidence (38 articles) and landmark papers were synthesized in the review. Study identification, screening, eligibility, and inclusion are summarized using a PRISMA-style flow diagram in accordance with the PRISMA 2020 reporting guidelines (Figure 1).

## 3. Etiology

Spondylolysis is a defect or stress fracture in the pars interarticularis, a small bony segment between the superior and inferior articular processes of the vertebra. It most commonly affects the lower lumbar spine, particularly the L5 vertebra, which accounts for up to 95% of cases, followed by the L4 vertebra, with recent studies demonstrating upper lumbar lesions may occur more than previously reported [7]. This distribution is attributed to the L5 vertebra’s location at the transition point between the mobile lumbar spine and the relatively fixed sacrum, making it more susceptible to mechanical stress [8,9]. The condition results from repetitive mechanical loading, especially during activities requiring spinal hyperextension and rotation, leading to stress fractures. Microtrauma accumulates in the pars interarticularis, and when the bone’s remodeling capacity is exceeded, fractures develop.

The risk factors for spondylolysis are multifactorial, encompassing genetic, mechanical, and lifestyle-related contributors. Genetic predispositions, such as a family history of spondylolysis and polymorphisms in genes affecting collagen synthesis and bone density, increase susceptibility [10]. Mechanical factors, including repetitive spinal hyperextension, axial loading, and rotational forces, are significant contributors, especially in athletes involved in sports like gymnastics, diving, wrestling, football, and, more generally, single sport participation at the exclusion of others [7,11]. A recent systemic review meta-analysis including nine studies and 835 patients reported the incidence of lumbar spondylolysis in athletes to be 41.7% with considerable variance among age and gender [9]. Recent studies in adolescent baseball players found lumbar lordosis to be more commonly found in those with spondylolysis compared to those without [12] with other associated clinical factors including pain for greater than 4 weeks, back pain that interferes with running, and pain that began unilaterally [13]. Pitching and batting with the dominant hand is more likely associated with contralateral side lesions in baseball players underscoring the importance of considering dominance and sport specific movements in evaluation of athletes [14]. Another study demonstrated pain with extension, difference between active and resting pain greater than 3/10, and male sex as the three patient characteristics that may aid in ruling out active spondylolysis when negative [15]. A study of soccer players demonstrated an association with the development of extension-based lumbar pain from an asymptomatic stress reaction of the pedicle and increased iliopsoas muscle tightness of the kicking leg compared to the supporting leg [16]. Another study described sacral anterior tilt, immature lumbar epiphyses, and hamstring tightness as risk factors for bilateral lumbar bone stress injury [17]. Training factors such as frequency, training duration, and pain-inducing occurrences may also influence development on spondylolysis [18]. Lifestyle factors also play a role; intense physical activity during growth spurts in adolescence is a notable risk [8]. On the other hand, sedentary behavior may indirectly contribute by reducing bone strength and resilience.

Spondylolysis has an estimated prevalence of 3–7% in the general population, but this increases to 11–15% among individuals participating in high-impact sports [4]. It is most commonly diagnosed in adolescents aged 10–15 years, coinciding with periods of rapid skeletal growth and peak sport participation [19]. Males are more frequently affected than females, with a male-to-female ratio of approximately 2:1, potentially due to higher participation in high-risk activities and anatomical differences in spinal curvature and pelvic tilt [9].

## 4. Diagnosis

Spondylolysis commonly affects L5 on S1. Patients with spondylolysis are commonly asymptomatic. Manifestations of symptoms include recurring low back pain, which is increased with activity and exacerbated by lumbar hyperextension, and possibly associated with radicular components. The pain that is experienced ranges mild to severe in intensity and is commonly described as a dull, aching pain in the lower back, and gluteal and posterior thigh area. Neurological signs and symptoms are not always present. When present, it is likely associated with multiple spondylolysis sites or degenerative changes in the neuroforamina leading to spinal nerve impingements. Through examination, neurological symptoms demonstrate increased lumbar lordosis, hamstring tightness, reduced trunk range of motion, tenderness to palpation over fracture site, a positive stork test, and or positive facet loading test. Notably, radicular symptoms can be present but are uncommon [20]. Unfortunately, despite a high prevalence in adolescent athletes, one single-center retrospective study found that the average time from symptom onset to initial presentation was 24 weeks and from initial presentation to diagnosis was 15 weeks with the average non-orthopedic provider taking 24 weeks longer to make the diagnosis [6].

The hyperextension test is commonly used as a physical examination for aiding in findings of early-stage spondylolysis (ESS), yet it has demonstrated that its association is not deemed significant. Clinical indications for EES are demonstrated through a negative result of hyperflexion in the fingertip-sized pain area and low back pain on the specific painful side [21]. Other diagnostic methods include the use of computed tomography (CT), the gold standard [19], single photon emission computerized tomography (SPECT), ultrasound (US), magnetic resonance imaging (MRI) and electromagnetic radiation (X-ray). A positive Doppler effect on the US is effective at screening for very early and early-stage spondylolysis in adolescents [22]. Advanced imaging modalities such as MRI, CT, and SPECT can be more sensitive than plain X-rays for diagnosing spondylolysis [19]. A recent MRI study utilizing deep learning technology demonstrated improved identification of pars interarticularis defects, which could be used to particularly improve the diagnosis of isthmic spondylolysis with further research ongoing [23].

In adolescents and young adults, the diagnosis of spondylolysis can be the result of varying symptoms specific to the bony injury, the compensatory symptoms, postural abnormalities, and movement dysfunctions. The clinical evaluation of a patient’s posture, spinal alignment, active range of motion (AROM), gait while walking and running, palpation of muscle tenderness, tone and step of deformities at the spine, movement analysis, lower body flexibility, motor control of the abdominal muscles, manual muscle testing of the lower extremities and trunk, and, lastly, neurological evaluations of L5 can be extremely effective in understanding the contributing factors leading to a spondylolysis diagnosis [19]. Utilizing models such as the Sahrmann’s Kinesiopathology Model of Movement (KMP) can also be beneficial when treating athletes with spondylolysis. Additionally, hip musculature tightness at the hamstring and hip flexors, increased muscle tone, painful spine AROM, and inhibition of the lumbopelvic girdle muscles are associated symptoms in patients diagnosed with spondylosis [24].

## 5. Management

Conservative treatment includes a period of rest, physical therapy targeting the deep abdominal muscles, lumbar multifidi, hamstrings, and flexion-based isometric back strengthening, and optionally bracing [8,11,25,26]. There is debate in the literature over the merits of bracing [27]. Multiple studies have shown that bracing may be beneficial for pain control, but long-term outcomes are similar [28]. Another potential benefit is that athletes are able to return to sport in a brace within 4–6 weeks [29]. Hard braces such as the Boston Overlap Brace are preferred over soft corsets because of the improved bone healing of the pars interarticularis [28]. Soft corsets do not restrict the spine enough to prevent the hyperextension of the pars interarticularis, which can lead to increased axial loading. Conservative treatment is best in adolescents with early-stage defects, i.e., hard bracing has been shown to lead to complete bone reunion of the pars interarticularis at 3 months [28]. To date, there are still no studies directly comparing the treatment of spondylolysis with and without bracing and it is argued that although studies have demonstrated radiographic benefit of bracing, it has not shown clinical benefit in achieving pain-free motion [27].

In terms of pharmacological intervention, spondylolysis can be treated with nonsteroidal anti-inflammatory drugs, e.g., ibuprofen and naproxen sodium. Corticosteroids may be used in some cases, e.g., prednisone, and may be injected in chronic cases and cases where severe pain is present. Traditionally, chronic pars defects have been regarded as benign and self-limiting, but a recent study demonstrated association with chronic pars interarticularis lesions and dorsiflexion and plantarflexion weakness with progression to chronic nerve injury [30]. Muscle relaxants may be used to treat spondylolysis, e.g., cyclobenzaprine. Antiseizure drugs and antidepressants can be used to relieve pain as well [31]. It should be noted that there are studies to suggest NSAID has been correlated with delayed healing and an increased risk of stress fractures, but to our knowledge no specific studies have been performed in spondylolysis [32].

As for physical therapy, strengthening the muscles of the lower lumbar spine and the core is ideal. A recent study demonstrated lumbar paraspinal muscle and psoas muscle ratio differences between acute lumbar spondylolysis and non-specific low back pain [33]. Flexion and extension exercises have been shown to improve pain and generate a return to activity [34]. Bracing immediately while performing flexion and extension exercises demonstrated improvements in pain as well as the healing of the pars interarticularis upon SPECT imaging [34]. Additionally, decreased recurrence rates were demonstrated by addressing thoracic spine mobility, hamstring, quadricep, and Achilles tightness, and core strength [35,36].

In severe cases of unilateral defects or bilateral defects, surgery may be indicated. Surgical treatment is indicated for patients that fail conservative treatment and is usually required in 9 to 15% of cases [11]. Indications for surgical intervention include pain, vertebral slippage, and neurological abnormalities [11]. Surgical treatment options include Scott wiring, buck repair, pedicle screw repair and the Morscher technique [8]. In terms of surgical outcome, pedicle screw repair and Buck repair had higher bone union rates with fewer complications than Scott wiring and Morscher technique [8].

The key to recovery for pars interarticularis injury is early detection and treatment. With early treatment it is possible for a reunion of the bone at the pars interarticularis with favorable outcomes, with conservative management in the majority of cases.

## 6. Complications and Prognosis

Complications from spondylolysis depend on the severity of the injury of the pars interarticularis with lower union rates with more advanced stages and in bilateral cases [37,38,39]. Recent radiographic studies of isolated spondylolysis demonstrated a high healing rate of 81.5% with conservative treatment and such healing demonstrated radiographically was associated with higher odds of symptomatic resolution and return to play [40]. Unilateral defects of the pars interarticularis can progress with further injury into a bilateral defect from further hyperextension and axial loading. A bilateral defect can progress into the slippage of the vertebrae, causing spondylolisthesis [28]. Spondylolysis can be categorized as isthmic, dysplastic, degenerative, traumatic, and pathologic [34]. Isthmic spondylolysis is injury to the pars interarticularis, which, as mentioned above, can lead to slippage of the vertebral body over the adjacent vertebrae, i.e., spondylolisthesis. Dysplastic spondylolysis is observed when there is abnormal formation of the spine at birth, particularly of the facets. Degenerative spondylolysis occurs with cartilage degeneration at the joints of the spine, i.e., arthritis. Traumatic spondylolysis occurs with trauma to the spine from physical forces that injure the pars interarticularis and may force the vertebrae out of alignment. Finally, pathologic spondylolysis is observed from co-occurring bone diseases, e.g., osteoporosis or tumors of the spine.

Long-term outcomes of spondylolysis depend on the severity of the illness, with many experiencing favorable outcomes with nonsurgical treatment. However, when the injury is severe, surgical intervention may be an option when conservative treatments fail.

## 7. Discussion

Reviewing the recent literature on spondylolysis reveals key patterns and findings related to the etiology, diagnosis, management, and prognosis of the condition. Studies like those from Tsai et al. [8] and Li et al. [3] emphasize that spondylolysis predominantly affects the L5 vertebra, with repetitive loading and hyperextension playing critical roles in injury development. Epidemiological data also corroborates this finding, highlighting a higher prevalence in athletes and males, aligning with the reports of Sundell et al. [5] and Fredrickson et al. [4]. Additionally, L5 lesions are associated with a higher non-union rate than non-L5 lesions, with the progressive stage being more associated with non-union on the main side [41,42]. However, there are disparities in the rates of diagnosis, with non-orthopedic providers reporting a much longer delay compared to orthopedic specialists, as observed by Nielsen et al. [6], indicating a gap in awareness and training among primary care providers.

The effectiveness of conservative treatments such as relative rest and physical therapy, as noted by Sairyo et al. [28] and Garet et al. [34], aligns with improved outcomes when diagnosed early, with the debate over bracing still limited by a lack of studies involving a head-to-head comparison of return to play, though long-term studies seem to demonstrate similar outcomes. A study utilizing 3D spine kinematics during activities of daily living demonstrated decreased thoracic and total lumbar spine extension in various activities that provided evidence that bracing reduces stress on the pars interarticularis, thus facilitating pain reduction and patients’ return to sports activities [43]. Studies also show that surgical intervention is necessary for a small proportion of patients, particularly those with severe cases or failure to respond to conservative treatments [8]. Outside of conventional approaches, there are studies of endoscopic and percutaneous direct approaches being successful and minimizing muscle and soft tissue dissection, reducing blood loss, and promoting early recovery and mobility [44,45]. This finding underscores the importance of early detection and personalized treatment strategies.

The clinical implications of these findings are significant for both diagnosis and treatment. The early identification of spondylolysis can lead to improved outcomes with conservative management, especially in adolescents who have a higher likelihood of complete bone healing. The use of hard braces, external bone stimulators, and physical therapy are effective in managing the condition, especially in the early stages, with a recent study showing excellent outcomes [46]. However, the delay in diagnosis and the lack of awareness among non-orthopedic practitioners pose challenges for timely intervention, which could prevent the condition from progressing to more severe stages like spondylolisthesis. Algorithms have been developed to help identify pediatric low back pain that may require advanced imaging with factors including lumbar lordosis angle, days after symptom onset, body mass index, and lumbosacral joint angle [47]. Still, there is variability in management, particularly with respect to bracing, PT, return to sport restrictions, and timing [48]. A small prospective study utilized an immediate functional progression program with a median return to sport participation of 2.5 months with a 92% success rate, which warrants future study into functional progression protocols [49]. Additionally, the effectiveness of physical therapy and bracing may vary across patients, and more tailored approaches are needed.

The limitations of current research include the limited longitudinal data on the long-term effects of conservative treatments including NSAIDs on healing times and the lack of consensus bracing and optimal treatment protocols for various stages of the condition. Additionally, while advanced imaging techniques like MRI and CT scans are highly sensitive, their cost and accessibility may limit their use in some populations, although commonly utilized in pediatric low back pain [50].

## 8. Future Directions

There are several gaps in the current research on spondylolysis that need to be addressed. One major area is the need for more long-term studies that evaluate the outcomes of conservative management over the course of an athlete’s career, particularly in sports where repetitive loading is frequent. Additionally, there is a lack of consensus regarding bracing, so future research could compare head-to-head trials of bracing compared with non-bracing to look for differences in return to pain free sport and fracture healing. Another gap lies in understanding the long-term effects of NSAID use and the prevalence of and fracture healing in spondylolysis.

Non-invasive, non-ionizing screening methods, such as ultrasound, show promise, but more research is needed to validate their sensitivity and specificity compared to gold-standard techniques like CT or MRI, which may potentially facilitate identifying more efficient, cost-effective diagnostic approaches and algorithms based on the laterality of symptoms and findings, the duration of symptoms, and symptom severity, as well as comparing the long-term outcomes of spondylolysis treated via sonographic diagnosis compared to the gold standard.

The emerging treatments for spondylolysis, such as the use of regenerative medicine (e.g., platelet-rich plasma or stem cell therapy), hold potential for improving recovery and reducing the need for surgery. Technological advances in imaging, such as functional MRI and enhanced SPECT scanning, may allow for the more accurate and earlier detection of the condition, enabling better-targeted interventions. Additionally, wearable technology and motion sensors could be integrated into rehabilitation protocols to monitor real-time progress, ensuring that exercises are performed correctly and consistently to prevent further injury.

## 9. Conclusions

Spondylolysis is a common yet often underdiagnosed condition, particularly in young athletes engaged in sports that place repetitive stress on the spine. Early detection and management, including physical therapy, are crucial to successful outcomes. However, delays in diagnosis, particularly by non-specialist providers, can lead to more severe complications like spondylolisthesis.

The clinical relevance of spondylolysis cannot be overstated given its prevalence in the adolescent athletic population, as it affects both athletic performance and long-term spinal health. Continued advancements in diagnostic techniques and treatment strategies, including emerging therapies and technologies, hold promise for improving both the early detection and management of spondylolysis. Moreover, future research addressing the biomechanical and environmental factors contributing to spondylolysis will further refine prevention and intervention strategies. Ultimately, a multi-disciplinary approach involving primary care, physical therapists, and non-operative and operative orthopedic providers is essential for improving outcomes and minimizing the long-term impact of this condition.

## Data Availability

No new data were created or analyzed in this study. All data supporting the findings of this review are derived from previously published articles cited in the reference list.
